# Rapidly progressive anti-GBM disease secondary to long-standing rheumatoid arthritis: a case report and literature review

**DOI:** 10.3389/fimmu.2025.1661117

**Published:** 2025-10-17

**Authors:** Ji Li, Jing Zhang, Sheng-Guang Li, Qian Guo, Jing Xu, Lina Zhang, Yadan Zou, Ting Long, Ruohan Yu, Yanfeng Zhang

**Affiliations:** ^1^ Department of Rheumatology and Immunology, Peking University International Hospital, Beijing, China; ^2^ Department of Rheumatology and Immunology, Peking University Shougang Hospital, Beijing, China

**Keywords:** rheumatoid arthritis, anti–glomerular basement membrane disease, rapidly progressive glomerulonephritis, autoimmune disease, case report

## Abstract

**Background:**

Long-standing rheumatoid arthritis (RA) complicated by anti–glomerular basement membrane (anti-GBM) disease is exceptionally rare.

**Case:**

A 71-year-old man with long-standing seropositive RA developed a rapidly progressive glomerulonephritis due to anti-GBM disease, without any known drug triggers. Despite plasmapheresis (therapeutic plasma exchange), corticosteroids, and low-dose cyclophosphamide, he remained dialysis-dependent; RA activity was subsequently controlled with tocilizumab. Complications included *Stenotrophomonas maltophilia* pneumonia, COVID-19 and cytomegalovirus infection, and he died of pneumonia eight months after diagnosis.

**Conclusion:**

This case highlights the need for early serological testing for anti-GBM disease in RA patients with unexplained hematuria/proteinuria and for immunosuppressive therapy mindful of infection risk. Additionally, our literature review identified only ten reported cases of RA with anti-GBM disease, highlighting the rarity of this condition.

## Introduction

Rheumatoid arthritis (RA) is a common systemic autoimmune disease that only infrequently causes severe glomerulonephritis. Renal involvement in RA has been documented in approximately 1–5% of patients, usually stemming from medication toxicity (e.g., NSAIDs, gold, penicillamine) or secondary amyloidosis rather than primary vasculitis (1, 2). In contrast, anti-glomerular basement membrane (anti-GBM) disease is an exceptionally rare cause of rapidly progressive glomerulonephritis (annual incidence ~0.6–1.8 cases per million) (3, 4). Without prompt intervention, anti-GBM disease can quickly progress to irreversible end-stage kidney disease (ESKD) and can be potentially fatal. True anti-GBM disease occurring secondary to RA is extraordinarily uncommon—only a handful of cases have been reported—yet when present it poses significant diagnostic and therapeutic challenges.

Here we describe a rare case of anti-GBM disease secondly to a long-standing seropositive RA in a 71-year-old man. Notably, this occurred without typical medication triggers. The presentation was an RPGN that progressed to irreversible kidney failure despite aggressive therapy. We also review the existing literature on RA with anti-GBM disease to explore potential pathogenic links (such as medication-induced immune dysregulation and intrinsic autoimmune propensity) and discuss the complexities of management in this scenario. This case underscores the need for a high index of suspicion for anti-GBM disease in RA patients with unexplained renal signs, as early recognition and intervention are critical for optimizing outcomes.

## Case presentation

A 71-year-old male with a 30-year history of poorly controlled seropositive RA was admitted in May 2024 with severe fatigue, anorexia, and oliguria that had progressed over two months. His RA had been characterized by chronic, erosive polyarthritis affecting the hands, wrists, elbows, shoulders, and ankles, leading to significant joint deformities and bilateral elbow contractures.

Past RA treatments included methotrexate (discontinued years prior due to gastrointestinal intolerance), low dose leflunomide (10 mg daily, ongoing), and intermittent use of *Tripterygium wilfordii* (a Chinese traditional herbal immunosuppressant). He also frequently took NSAIDs for pain management. Despite these therapies, his RA disease activity remained high, with persistent inflammation and deforming joint damage.

Routine laboratory tests five months before admission (December 2023) had already shown early renal abnormalities, specifically: proteinuria (+ on dipstick), gross hematuria (+++), and elevated inflammatory markers (C-reactive protein [CRP] 20.9 mg/L and erythrocyte sedimentation rate [ESR] 79 mm/hour). Unfortunately, these findings were overlooked at the time – they were attributed to a possible urinary tract infection or NSAID use, and no further nephrological evaluation was pursued. The patient did not report any urinary symptoms then, and follow-up was missed amid his ongoing RA issues. This represented a missed opportunity for earlier diagnosis.

By May 2024, the patient’s condition worsened dramatically. He developed marked fatigue, loss of appetite, and his urine output dwindled to <400 mL/day (oliguria). On May 16, 2024, laboratory tests showed a serum creatinine of 1038 µmol/L (baseline ~80 µmol/L in late 2023). Over just a few days, his creatinine spiked further to 1920 µmol/L by May 21. He also exhibited severe uremic symptoms (nausea, confusion) and electrolyte disturbances (potassium 6.3 mmol/L, sodium 125 mmol/L) consistent with AKI. Arterial blood gas revealed metabolic acidosis. He was anemic (hemoglobin 117 g/L, which later dropped further) and had profound hypoalbuminemia (20.9 g/L). Inflammatory markers were extremely elevated (CRP >186 mg/L, ESR >140 mm/hour), and NT-proBNP was >35,000 pg/mL, reflecting fluid overload and possible cardiac strain (**Table 1**).

**Table 1:** Key laboratory and imaging results at critical clinical stages of the present case (RA with anti-GBM disease).

Parameter	References	2023/12/8	2024/5/16	2024/5/21	2024/5/24	2024/6/12	2024/6/17	2024/6/21	2024/7/11	2024/8/28	2024/9/3
Hemoglobin (g/L)	**130-175**	155	117	110	73	84	90	88	121	122	120
Platelets (×10^9^/L)	**125-350**	224	337	395	119	129	46	58	115	175	138
WBC (×10^9^/L)	**3.5-9.5**	8.4	8.14	11.15	8.77	8.27	7.94	6.42 (Neu 79.5%)	9.97	8.7	9.07 (Neu 89.2%)
CRP (mg/L)	**≤10**	20.9	–	211	186.98	–	3.82	4.5	2.94	2.32	25.02
ESR (mm/h)	**0-15**	79	120	–	>140	–	–	–	2	14	–
Creatinine (μmol/L)	**59-104**	80	1038	1920	662	494	659	597	479	946	945
Anti-GBM antibody (CU)	**<20**	–	–	–	655.5	84.9	–	55	36.6	12.6	6.7
Anti-CCP (U/mL)	**0-17**	–	–	–	>500	–	–	–	–	–	–
Rheumatoid Factor (IU/mL)	**0-20**	160	–	–	92.9	–	–	–	–	–	–
Interleukin-6 (pg/mL)	**0-7**	–	–	573.5	222.5	–	–	7.34	3.77	–	–
Procalcitonin (ng/mL)	**≤0.05**	–	–	4.27	2	–	–	0.34	2.53	1.82	0.57
NT-proBNP (pg/mL)	**≤125**	–	–	>35000	>35000	–	–	26019	14978	2800	–
Albumin (g/L)	**40-55**	–	33.6	27.1	20.9	29.1	35.4	32.6	35.8	40.6	–
Calcium (mmol/L)	**2.11-2.52**	–	1.83	1.57	1.81	–	1.92	1.86	1.96	2.17	–
Phosphate (mmol/L)	**0.85-1.51**	–	2.12	4.06	1.71	–	1.62	1.26	1.24	1.8	–
Potassium (mmol/L)	**3.5-5-3**	–	5.7	6.94	3.29 (after dialysis)	3.85	3.87	3.5	4.03	4.65	4.3
Sodium (mmol/L)	**137-147**	–	132	128	136	143	145	142	135	136	136
D-dimer (ng/mL)	**≤250**	–	–	1685	990	385	370	392	294	–	522
Fibrinogen (mg/dL)	**200-400**	–	–	986	847	189	151	157	169	–	466
Lung CT findings		–	–	Interstitial fibrosis, inflammation	Infection	–	–	–	Infection resolved	Worsened interstitial inflammation	Severe pulmonary inflammation
Joint Ultrasound		–	–	–	–	–	–	Active synovitis, effusion, erosions	–	–	–
Pathogens		–	–	–	Stenotrophomonas maltophilia (+++), fungi (+)	–	–	–	–	–	SARS-CoV-2 (+), CMV-DNA (+)

WBC, white blood cell count; Neu, neutrophils; ESR, erythrocyte sedimentation rate; GBM, glomerular basement membrane; CCP, cyclic citrullinated peptide; NT-proBNP, N-terminal pro–B-type natriuretic peptide; HD, hemodialysis; PEX, plasmapheresis; CT – computed tomography; –: not measured.

The bolded values in the table represent the reference ranges for each laboratory parameter, as established by our institutional laboratory. These ranges are provided to facilitate the interpretation of the patient's results in clinical context.

A chest computed tomography (CT) scan on admission showed bilateral interstitial infiltrates and fibrosis, suggestive of RA-associated interstitial lung disease (ILD) versus infection (no discrete lobar consolidation was seen). There were no radiological signs of diffuse alveolar hemorrhage (such as widespread ground-glass opacities), and the patient did not report hemoptysis. Given his critical state, we did not perform bronchoscopy; however, the absence of hemoptysis and the pattern of CT changes made anti-GBM disease-related pulmonary hemorrhage unlikely. The interstitial changes were attributed to chronic RA lung involvement, with superimposed pneumonia also considered. Additionally, a renal ultrasound at admission showed no evidence of obstruction (no hydronephrosis), making a post-renal cause of hematuria unlikely. The urinalysis findings (including red blood cell casts) further supported a glomerular source of bleeding rather than a urologic cause.

On admission, the patient was in critical condition. He was hypotensive (BP 85/50 mmHg) and tachypneic, with signs of fluid overload (marked bilateral leg edema) and uremic encephalopathy (asterixis and confusion). He had basal crackles on lung auscultation (consistent with fluid/inflammatory infiltrates), but no frank pulmonary hemorrhage. Joint exam showed active synovitis in multiple joints, consistent with an ongoing RA flare (he had painful swelling in the wrists, knees, and ankles despite his advanced joint deformities).

Emergency hemodialysis was initiated on admission to manage AKI and metabolic derangements. Empiric broad-spectrum antibiotics were started (meropenem) for possible sepsis. We also obtained extensive immunological tests given the unexplained RPGN. Strikingly, results revealed a very high titer of circulating anti-GBM antibodies (655.5 Chemiluminescence Units; normal <20 CU). Anti-neutrophil cytoplasmic antibodies (ANCA) were negative. RA serologies remained strongly positive (anti-CCP >500 U/mL, rheumatoid factor 92.9 IU/mL), reflecting active disease. Given the combination of oliguric RPGN and a high anti-GBM antibody titer, a diagnosis of anti-GBM disease was made. No renal biopsy was performed – the patient was hemodynamically unstable and coagulopathic, and he (and his family) did not consent to an invasive biopsy. We acknowledge that lacking histology is a limitation; however, the clinical presentation and serology were considered confirmatory in this emergency setting.

Microbiological studies aided in clarifying the lung findings: sputum cultures grew *Stenotrophomonas maltophilia* (a multi-drug-resistant Gram-negative organism) at heavy growth, and fungal cultures grew *Candida* species. These results indicated a severe hospital-acquired pneumonia with fungal co-infection. The patient was started on targeted antimicrobial therapy (intravenous trimethoprim-sulfamethoxazole for *S. maltophilia*, which also served as *Pneumocystis jirovecii* prophylaxis, and an echinocandin antifungal for *Candida* coverage). We also implemented prophylactic measures appropriate for his immunosuppressed state – for example, trimethoprim-sulfamethoxazole was continued prophylactically to prevent *Pneumocystis* pneumonia and other opportunistic infections, and fluconazole was given to prevent fungal thrush. Infection control was a major ongoing concern throughout his treatment.

A multidisciplinary team (nephrology, rheumatology, pulmonology, intensive care) coordinated the next steps. We initiated plasmapheresis on hospital day 2, given the classic indication for anti-GBM disease. He underwent a total of eight plasmapheresis sessions (replacing plasma with albumin) over the next two weeks. Concurrently, we started high-dose corticosteroids (initially IV methylprednisolone 40 mg daily, then tapering to oral prednisone 40 mg/day after one week). For additional immunosuppression, we carefully introduced cyclophosphamide – however, considering the patient’s advanced age and active infections, we used a cautious low-dose regimen (50 mg every other day orally) instead of the standard high-dose protocol. This adjusted dosing was chosen to balance treating the anti-GBM disease with avoiding further immunocompromise.

**Figure 1:**
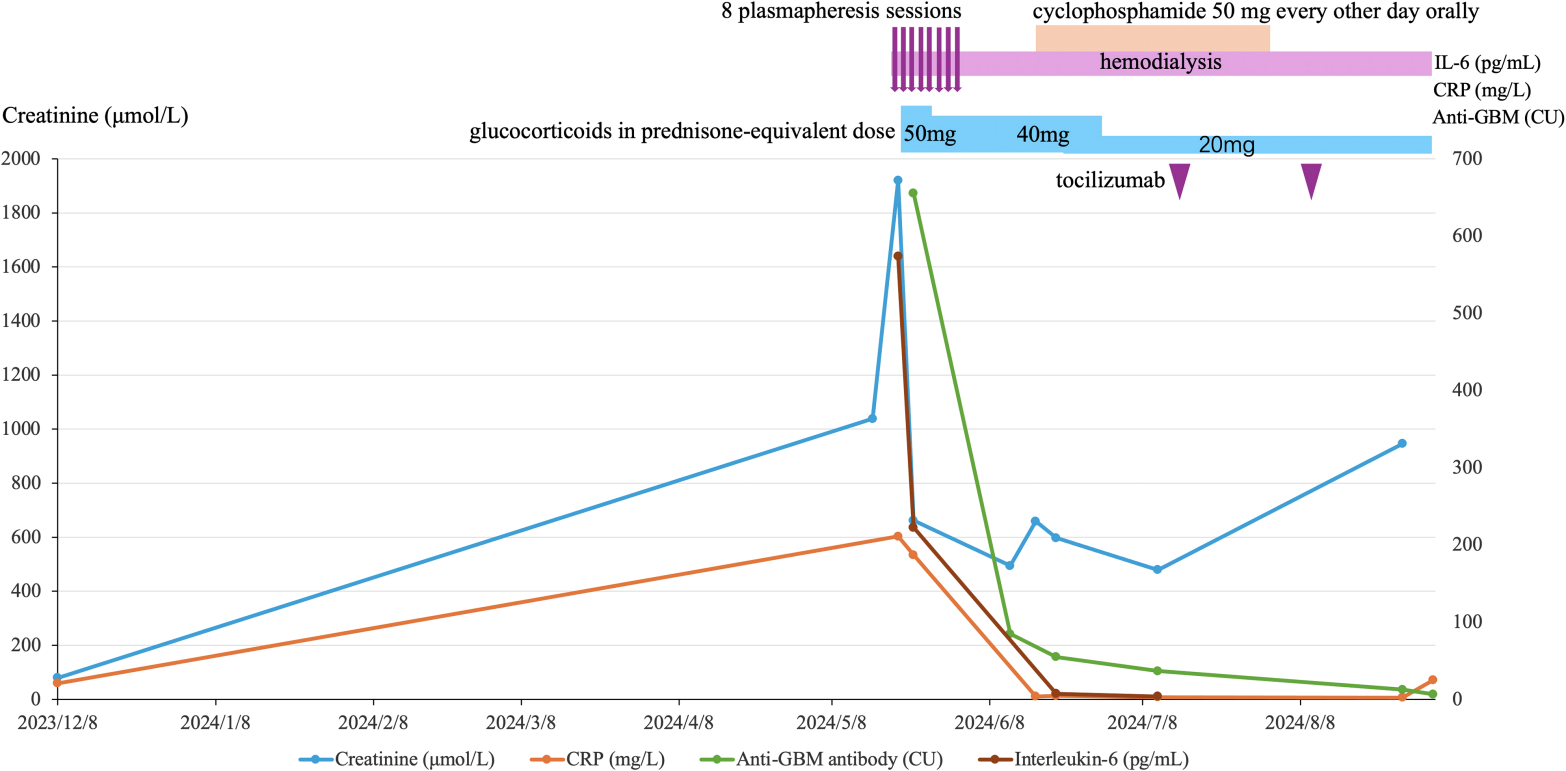
Monthly timeline of key laboratory markers and therapies in anti-GBM disease secondary to long-standing rheumatoid arthritis. From the first evidence of renal injury (Dec 2023) to Aug 2024, the figure plots four markers: serum creatinine (blue, left y-axis), anti-GBM antibody titer (green, right y-axis), CRP (orange, right y-axis), and IL-6 (brown, right y-axis). Treatment overlays above the plot denote 8 plasmapheresis sessions (mid–late May 2024), ongoing hemodialysis, glucocorticoid taper (prednisone-equivalent 50 mg → 40 mg → 20 mg), a short course of oral cyclophosphamide (50 mg every other day), and two tocilizumab infusions. After initiation of plasmapheresis and immunosuppression, anti-GBM, CRP and IL-6 declined sharply, whereas creatinine peaked and then only partially improved, remaining elevated in keeping with dialysis dependence. Curves connect measured time points to illustrate trends.

Remarkably, after about 6 plasmapheresis sessions, the patient’s anti-GBM antibody titer dropped from 655.5 CU to 84.9 CU. After the full 8 plasmapheresis (by early June 2024), the titer further declined to 55 CU (just above the upper limit of normal). Given this near normalization of anti-GBM antibody levels and economic concern, plasmapheresis was concluded after the eighth exchange to minimize additional immunosuppressive exposure and infection risk in this elderly patient. (At follow-up visits in July, August, and September 2024, his anti-GBM antibody titers further decreased to 36.6, 12.6, and 6.7 CU, respectively, as shown in **Table 1** and **Figure 1**). This serological response indicated that our therapy was successful in removing circulating autoantibodies. Clinically, the patient’s pulmonary status improved: follow-up chest imaging in June showed resolution of the pneumonia infiltrates, although the underlying interstitial lung fibrosis had progressed (likely due to RA-ILD). Importantly, he never developed any signs of alveolar hemorrhage during hospitalization, suggesting our timely interventions may have averted the pulmonary manifestations of anti-GBM disease.

Despite clearing the anti-GBM antibodies from the circulation, the renal damage was already irreversible at presentation. The patient remained dialysis-dependent; his urine output did not recover (consistent with advanced RPGN and essentially complete ESKD by the time therapy began). We continued intermittent hemodialysis three times per week as chronic renal replacement therapy.

By mid-June, as the acute phase of the anti-GBM disease stabilized, we faced active RA issues. With steroid tapering (we reduced prednisone to 20 mg/day by late June), the patient developed a flare of his arthritis – swelling and pain recurred in multiple joints (particularly knees and shoulders). Considering his refractory RA and contraindications to some other therapies, we started tocilizumab, an IL-6 receptor inhibitor, for its efficacy in RA and relative safety in infection-prone patients. After obtaining clearance from the infectious disease team, tocilizumab 8 mg/kg IV was administered (with doses planned every 4 weeks). This led to a notable improvement in his joint symptoms and inflammatory markers over the next month. We also administered local therapy (intra-articular steroid injections to his shoulders and knees) to provide additional relief during the flare.

During the course of treatment, we were vigilant about prophylaxis and monitoring for opportunistic infections. Despite these efforts, the patient’s recovery was complicated by several serious infections due to his prolonged immunosuppression: in July 2024 he tested positive for COVID-19 (with moderate pneumonia), and shortly thereafter he developed reactivation of cytomegalovirus (CMV) (confirmed by PCR, with CMV viremia and hepatitis). He was treated with supportive care and antivirals (remdesivir for COVID-19, then ganciclovir for CMV). Cyclophosphamide was promptly discontinued by this time (after ~6 weeks of therapy) to help immune recovery, and we managed him with low-dose prednisone plus tocilizumab for RA thereafter. The patient initially recovered from both COVID-19 and CMV infections by August 2024, thanks to aggressive antimicrobial therapy and supportive ICU care.

Unfortunately, on 3 January 2025, approximately eight months after the anti-GBM diagnosis, the patient suffered a new episode of severe pneumonia (unrelated to COVID-19, likely bacterial). Despite broad-spectrum antibiotics and maximal supportive therapy, his condition deteriorated rapidly, and he passed away in the hospital due to respiratory failure. The cause of death was determined to be overwhelming pneumonia on a background of immunosuppression and end-stage renal disease.

This case timeline is visually summarized in **Figure 1**. It underscores how an initial oversight of renal warning signs led to a cascade of critical illness. Despite timely use of plasmapheresis and immunosuppression once diagnosed (which potentially prevented pulmonary hemorrhage), the delay resulted in permanent dialysis dependence and set the stage for fatal infectious complications. Earlier recognition and intervention might have altered the outcome, highlighting a crucial lesson for clinicians managing complex patients with autoimmune diseases.

## Discussion

**Figure 2:**
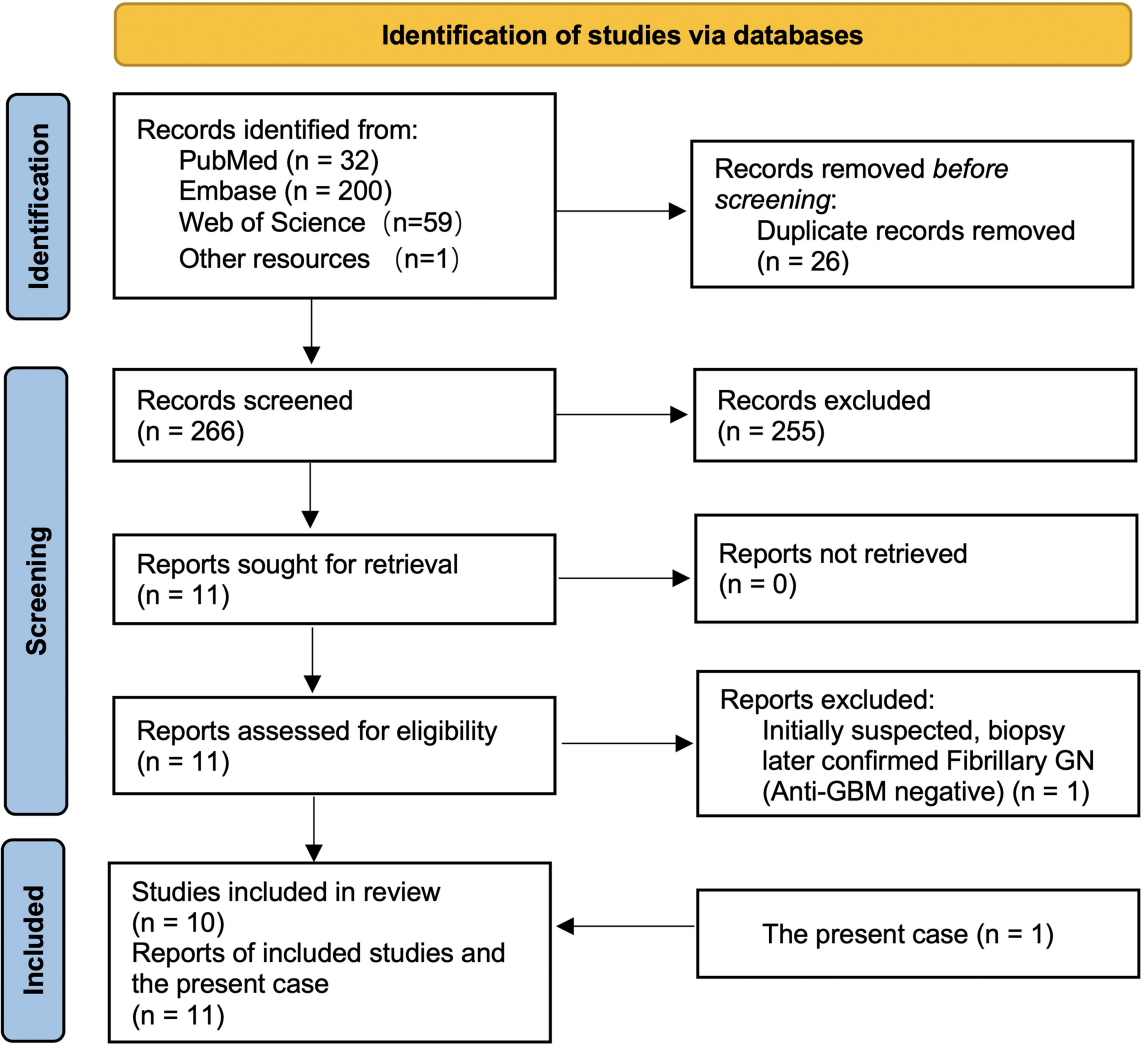
PRISMA flow of study selection for RA complicated by anti-GBM disease. A database search (PubMed = 32, Embase = 200, Web of Science = 59, other sources = 1) identified 292 records. After removing 26 duplicates, 266 records were screened and 255 excluded. Eleven full-text reports were assessed (none unretrieved); one was excluded after biopsy confirmed fibrillary GN with negative anti-GBM. Ten studies were included in the review, and together with the present case (n = 1) yielded eleven reports in total.

The coexistence of RA and anti-GBM disease is exceedingly rare. Through our updated review, we identified eight previously reported RA with anti-GBM cases worldwide (six full reports and two conference abstracts). We conducted a search of PubMed, Embase, and Web of Science, together with forward/backward citation tracking and other open resources (e.g., CrossRef/Google Scholar), from inception to September 2025, which identified 292 records (PubMed n = 32; Embase n = 200; Web of Science n = 59; other resources n = 1). After removing 26 duplicates, 266 records were screened; eleven reports underwent full-text assessment, one was excluded because the biopsy confirmed fibrillary GN with negative anti-GBM serology, and ten studies were included, as shown in **Figure 2**. Each reported case displayed unique features, underscoring substantial heterogeneity in this overlap (5–14). Our patient adds another documented instance to this limited body of evidence and highlights several points that may refine clinical understanding.

### Medication triggers vs. intrinsic autoimmunity

Among the ten RA cases, five appear drug-related. One followed D-penicillamine (Peces et al., 1987) (5); one followed leflunomide (Bruyn et al., 2010) (8); and three occurred after adalimumab therapy for RA — reported by Nishimura et al (2012) (9), Sánchez et al (2015) (10), and Heron et al (2020) (11) (see **Table 2**). Outside RA, Al-Chalabi et al (2021). described an anti-GBM disease in a patient with *psoriatic arthritis* receiving etanercept (15). Even so, TNF-inhibitor–associated reports remain very few relative to the widespread use of these agents, and experimental models suggest TNF-α blockade may even attenuate anti-GBM nephritis (16–18); therefore, any causal link should be interpreted with caution. By contrast, our patient had no exposure to penicillamine or any TNF inhibitor. He had taken leflunomide and intermittently *Tripterygium wilfordii*, with occasional NSAIDs; given that leflunomide has been linked to anti-GBM disease in a prior RA case, a drug contribution cannot be excluded but remains unproven.

**Table 2:** Clinical characteristics, diagnosis, treatment, and prognosis of patients with rheumatoid arthritis complicated by anti-GBM disease.

Author (ref.)	Year	Gender	Age	Initial disease	Anti-GBM antibody confirmed	Main GBM presentation	Drug-related?	Diagnosis confirmation	Treatment measures	Prognosis
Peces et al. (5)	1987	Female	51	RA	Yes	Acute renal failure, pulmonary hemorrhage, proteinuria	Yes (D-penicillamine)	Anti-GBM antibody positive; Renal biopsy: Focal necrotizing crescentic glomerulonephritis.	Pulse steroids, cyclophosphamide	Improved renal function, anti-GBM negative at discharge
Hiroshige et al. (6)	1997	Female	63	RA	Yes (with MPO-ANCA)	microscopic hematuria, proteinuria, and rapidly advancing renal dysfunction	No	Anti-GBM antibody positive; Renal biopsy: Diffuse crescentic glomerulonephritis (crescents in all glomeruli)	Plasmapheresis, steroids, cyclophosphamide, and heparin	progressive deterioration to end-stage renal disease despite treatment
Takeda et al. (7)	2009	Female	50	RA	Yes	Acute renal failure, pulmonary hemorrhage, nephrotic syndrome	No	Anti-GBM antibody positive; Renal biopsy: extensive crescentic GN plus minimal change nephrotic changes.	Plasmapheresis, steroids, hemodialysis	Improved but chronic kidney impairment remained
Bruyn et al. (8)	2010	Female	60	RA	Yes	Rapidly progressive GN with acute renal failure	Yes (leflunomide)	Anti-GBM antibody positive; Renal biopsy: Diffuse crescentic GN (all glomeruli affected).	Plasmapheresis, cyclophosphamide, steroids	Kidney failure irreversible, dialysis-dependent
Nishimura et al. (9)	2012	Female	69	RA	Yes (with MPO-ANCA)	acute RPGN with Cr ~401 μmol/L, microscopic hematuria.	Yes (adalimumab)	Serology tests, clinical presentation	Plasmapheresis, cyclophosphamide, steroids	Kidney failure irreversible, dialysis-dependent
Sanchez et al. (10)	2015	Male	73	RA	Yes	Acute renal failure, proteinuria, alveolar hemorrhage later	Yes (adalimumab)	Serology tests, clinical presentation	Plasmapheresis, dialysis, stopped Adalimumab	End-stage renal failure, dialysis-dependent
Heron et al (11).	2020	Male	63	RA	Yes	Rapidly progressive renal failure, proteinuria, hematuria	Yes (adalimumab)	Anti-GBM antibody positive; Renal biopsy: Severe crescentic GN (most glomeruli with cellular crescents).	Methylprednisolone pulse, plasmapheresis cyclophosphamide,	Dialysis-dependent (kidney transplant planned)
Komala et al. (12)	2021	Female	69	RA	Yes	Renal dysfunction, proteinuria, no hematuria	No	Anti-GBM antibody positive; Renal biopsy: Crescentic necrotizing glomerulonephritis.	Plasmapheresis, steroids, cyclophosphamide	Improvement of renal function
Cheng et al. (13)	2022	Female	62	RA	Yes (with MPO-ANCA)	progressive increase in serum creatinine	No	Anti-GBM antibody positive; Renal biopsy: extensive crescents with linear IgG on GBM.	Plasmapheresis, two rounds of IV methylprednisolone pulses (500 mg ×3 days each), cyclophosphamide, and continuous hemofiltration	Stabilized clinically; dialysis discontinued with partial renal function recovery
Kawamori et al. (14)	2024	Male	71	RA	Yes (with PR3-ANCA)	Mild renal impairment, proteinuria	No	Anti-GBM antibody positive; Renal biopsy: Membranous nephropathy (secondary to RA; subepithelial immune deposits, IgG_1_-dominant granular staining) with no crescentic GN features.	Intensified RA therapy	Remission of proteinuria
the present case	2025	Male	71	RA	Yes	Rapidly progressive renal failure, proteinuria, hematuria, interstitial fibrosis	No	Serology tests, clinical presentation	Plasmapheresis, steroids, cyclophosphamide, tocilizumab	End-stage renal failure, dialysis-dependent, died from infection

RA, rheumatoid arthritis; GBM, glomerular basement membrane; GN, glomerulonephritis; RPGN, Rapidly progressive glomerulonephritis; IV, intravenous. All patients were seropositive for RA (RF and/or CCP) except where not specified. All anti-GBM disease diagnoses were supported by serology (positive circulating anti-GBM antibodies) in addition to biopsy or clinical features. Outcomes are as reported at last follow-up in each case.

### Active RA and renal complications

Active RA itself can cause renal complications (e.g., secondary AA amyloidosis from chronic inflammation), and some RA treatments – notably long-term NSAIDs or certain disease-modifying drugs like gold or penicillamine – are known to have nephrotoxic effects (19, 20). These factors might contribute to mild chronic kidney impairment, but they would not typically provoke the kind of fulminant glomerulonephritis observed in our patient. Indeed, his AKI was far more severe than what NSAIDs or amyloid alone would usually cause, and it correlated with a very high anti-GBM antibody titer, indicating that anti-GBM disease was the primary culprit. This aligns more with other cases where no clear trigger was identified: for example, Takeda et al. (2009) documented idiopathic anti-GBM disease in an RA patient without any drug trigger or ANCA, suggesting that intrinsic immune dysregulation within RA could be enough to generate anti-GBM antibodies (7). Our case supports this idea – that long-standing RA itself, with chronic immune activation, might rarely set the stage for anti-GBM autoimmunity. Potential mechanisms discussed in the literature include genetic predisposition (e.g., certain HLA alleles confer risk for RA and other for anti-GBM) and epitope spreading from prolonged inflammation (where cryptic GBM antigens might be exposed during RA-related tissue damage, inciting a new autoimmune response). While speculative, these mechanisms underscore that RA patients – especially those with uncontrolled inflammation – could harbor an environment conducive to secondary autoimmune phenomena.

### Clinical variability and diagnostic challenges

The reported RA–anti-GBM cases have shown variable clinical presentations. Some, like Komala et al. (2021), had atypical features such as absence of hematuria or pulmonary hemorrhage, making diagnosis less straightforward (12). Another case by Kawamori et al. (2024) was double-positive for anti-GBM and ANCA and, interestingly, had a renal biopsy showing membranous nephropathy rather than classic crescentic GN. This underscores that histology can vary and overlap with other pathologies; it also emphasizes that a renal biopsy is invaluable when feasible, to delineate the pathology (something our case regrettably lacked due to the patient’s condition) (14).

Among the 11 cases in our series, 4 patients (36.4%) were ANCA-positive (1 PR3-ANCA and 3 MPO-ANCA), and only 3 cases (27.3%) exhibited pulmonary hemorrhage, whereas the remaining 8 cases (including the present case) had no lung hemorrhage, as summarized in **Table 2**. This trend is consistent with the meta-analysis by Huang et al., which reported a pulmonary hemorrhage rate of 32.6% in anti-GBM disease (3), indicating that roughly one-third of such patients develop this complication.

Our case presented as a pulmonary-renal syndrome in terms of multi-system involvement, but notably without alveolar hemorrhage. The patient’s respiratory findings were due to infection and RA-ILD rather than any anti-GBM disease–related pulmonary hemorrhage. We cannot overemphasize the importance of thoroughly evaluating such patients for lung hemorrhage: in our patient, careful assessment of imaging and clinical signs (with input from pulmonology) was crucial to confirm that the lungs were “innocent bystanders” apart from infection/RA, allowing us to focus on treating the renal crisis. Epidemiologically, most anti-GBM patients have renal involvement, and approximately two-thirds (≈67%) present without pulmonary hemorrhage, i.e., with a renal-limited course (3).

**Diagnostically,** our case teaches a cautionary lesson. The patient had early clues (hematuria, proteinuria) that were missed, illustrating how concurrent chronic disease (RA) can distract from recognizing a new, superimposed condition. We reflected on why the warning signs were overlooked: the patient’s mild symptoms in late 2023 did not prompt him to seek extra help, and his providers at the time attributed abnormal labs to benign causes (e.g., a possible UTI or NSAID effect) without pursuing nephrology consult or antibody testing. This represents an instance of anchoring bias – assuming everything is due to the known RA, thereby “anchoring” on that diagnosis and not fully evaluating out-of-character findings.

**Pathogenetically,** possible links between RA and anti-GBM disease include medication-induced immune dysregulation, genetic susceptibility (HLA alleles), and chronic RA with inflammation possibly exposing cryptic GBM antigens (21). However, detailed genetic studies remain lacking, and further research is needed to clarify the underlying mechanisms definitively.

### Prognosis and outcomes

Clinically, prognosis has varied significantly among reported cases, heavily influenced by the severity of initial renal dysfunction, pulmonary involvement, and infectious complications from immunosuppression. Earlier recognition, prompt serological and histological diagnosis, and aggressive plasmapheresis plus immunosuppression significantly influenced outcomes positively in reported cases (as noted in prior literature) (22).

### Management challenges – immunosuppression vs. infection

Treating anti-GBM disease requires aggressive immunosuppression (per standard practice, a combination of plasmapheresis, high-dose corticosteroids, and cyclophosphamide). In our patient, this collided with the reality of an elderly host prone to infections and an already immunocompromised state from RA. Our management had to be highly individualized in the absence of any precedent or specific guidelines for this overlap. We followed general principles from KDIGO for the anti-GBM treatment (22), but adjusted: for instance, using a reduced cyclophosphamide dose due to the active infection at presentation. Even with careful dosing and eventually discontinuing cyclophosphamide early, the patient developed severe opportunistic infections. This underlines a key point: there are no established treatment guidelines for RA with anti-GBM disease, so clinicians must balance competing risks on a case-by-case basis. In our discussion, we emphasize that multidisciplinary input (nephrology deciding how aggressively to immunosuppress, rheumatology managing RA therapy, infectious disease overseeing prophylaxis) is essential. The lack of formal guidelines is a gap – all reported cases (including the present case) had different therapeutic approaches and outcomes, which we summarize in **Table 2**. Until more cases are studied, treatment remains an art of customization. We have highlighted this to illustrate how we tailored our patient’s treatment (for example, plasmapheresis frequency was adjusted based on antibody levels; cyclophosphamide was tapered off early; tocilizumab was chosen for RA control to avoid potential triggers).

Our case also illustrates the delicate timing of introducing RA therapy after the acute anti-GBM illness. We started tocilizumab once the patient’s condition allowed (after initial infections were treated) to address his active RA. This likely improved his quality of life (relieving arthritis) and helped reduce steroid exposure, but it may have contributed to persistent susceptibility to infections (IL-6 inhibition can also impair host defenses). It’s hard to say whether withholding RA therapy would have changed the infection outcome, since uncontrolled RA itself can cause debility and perhaps increase infection risk (through high inflammation and the need for corticosteroids). This scenario highlights the conundrum that managing two active autoimmune diseases simultaneously is a tightrope walk, and decisions must be continuously re-evaluated as the patient’s status evolves.

### Prognosis

The prognosis in reported RA–anti-GBM cases has varied widely. Some patients (especially those with milder initial renal impairment or prompt treatment) survived with preserved kidney function. Others, like ours, had poor outcomes with dialysis dependence or death. A common theme is that the severity of initial renal dysfunction and any delays in therapy strongly influence outcomes. In our patient, by the time of diagnosis, his kidneys were virtually non-functional (creatinine ~1900 µmol/L, requiring immediate dialysis). Despite our aggressive measures halting further damage (and likely preventing pulmonary hemorrhage), we could not restore renal function. He remained on dialysis and ultimately succumbed to infection – an outcome that aligns with known observations that worse kidney function at diagnosis portends higher mortality and lower chance of renal recovery.

### Mechanistic considerations and immunogenetics

Chronic RA inflammation may facilitate epitope spreading, whereby tissue injury exposes cryptic GBM epitopes and breaks tolerance. Genetically, anti-GBM disease is strongly associated with *HLA-DRB1*15:01 (21), whereas RA is linked to “shared epitope” alleles such as *04:01 and *04:04 (23). These are distinct susceptibility alleles and should not be interpreted as a shared epitope across the two conditions. While a convergent impact on immune activation has been hypothesized, there is no evidence demonstrating a common antigen-presentation overlap between RA and anti-GBM. Complement and IL-6 pathways have been implicated in anti-GBM pathogenesis, supporting consideration of IL-6 blockade for RA control on a case-by-case basis; importantly, no causal link to TNF-α inhibitors has been established.

### Infection prevention and monitoring under dual immunosuppression

For patients requiring combined anti-GBM therapy (plasmapheresis + high-dose corticosteroids + cyclophosphamide) and concurrent RA control, evidence-based strategies include: *Pneumocystis* prophylaxis with trimethoprim–sulfamethoxazole; up-to-date vaccination (influenza, pneumococcal, COVID-19) before or between treatment cycles; baseline screening and periodic PCR monitoring for CMV in high-risk or lymphopenic patients; judicious antifungal use with de-escalation once cultures clear; catheter care and early removal/exchange; growth-factor support and nutritional optimization; and dose/interval tailoring of cytotoxic agents.

## Conclusion

In summary, this case underscores several key lessons. Clinicians caring for patients with rheumatoid arthritis should maintain a high level of vigilance for anti-GBM disease when otherwise unexplained hematuria or proteinuria is encountered. Once the diagnosis is established, plasmapheresis and immunosuppression need to be initiated as early as possible to maximize the chance of preserving renal function. At the same time, the coexistence of RA and anti-GBM disease demands an individualized approach that carefully balances the need for effective immunosuppression with the heightened risk of infection. Close collaboration between nephrology, rheumatology, and infectious disease specialists is essential to achieve optimal outcomes in such complex cases.

## Data Availability

The original contributions presented in the study are included in the article/supplementary material. Further inquiries can be directed to the corresponding author.
